# Excessive exogenous cholesterol activating intestinal LXRα-ABCA1/G5/G8 signaling pathway can not reverse atherosclerosis in ApoE^−/−^ mice

**DOI:** 10.1186/s12944-023-01810-6

**Published:** 2023-04-15

**Authors:** Xichao Yu, Xue Ding, Han Feng, Yunhui Bi, Yu Li, Jinjun Shan, Huimin Bian

**Affiliations:** 1 School of Pharmacy, Nanjing University of Chinese Medicine, Nanjing, 210023 China; 2 National Standard Laboratory of Pharmacology of Chinese Materia Medica, Nanjing University of Chinese Medicine, Nanjing, 210023 China; 3 Jiangsu Key Laboratory for Pharmacology and Safety Evaluation of Chinese Materia Medica, Nanjing University of Chinese Medicine, Nanjing, 210023 China; 4 Jiangsu Key Laboratory of Therapeutic Material of Chinese Medicine, Nanjing University of Chinese Medicine, Nanjing, 210023 China; 5 School of Medicine & Holistic Integrative Medicine, Nanjing University of Chinese Medicine, Nanjing, 210023 China; 6 Institute of Pediatrics, Jiangsu Key Laboratory of Pediatric Respiratory Disease, Nanjing University of Chinese Medicine, Nanjing, 210023 China; 7 Medical Metabolomics Center, Nanjing University of Chinese Medicine, Nanjing, 210023 China

**Keywords:** Exogenous cholesterol, Lipids metabolomics, Atherosclerosis, LXRα-ABCA1/G5/G8

## Abstract

**Background:**

The long-term excessive intake of exogenous cholesterol can lead to abnormally elevated blood lipid levels and induce cardiovascular and cerebrovascular diseases. However, the influence and relevance of exogenous cholesterol on plasma cholesterol components were still unclear, and the influence on intestinal lipid metabolism targets needs to be further explored.

**Methods:**

In vivo, the C57BL/6 + NF group and ApoE^−/−^ + NF group mice were fed a normal specific pathogen-free (SPF) diet; the ApoE^−/−^ + HF group mice were fed a high-cholesterol SPF diet. The plasma and jejunum tissue homogenate were obtained for non-targeted lipid metabolomics. The lipid droplets in tissues were observed by transmission electron microscope and oil red O staining. Jejunum tissue morphology was observed by HE staining. The kits were used to detect lipid content in plasma, tissues, intestinal contents, and cells. Western blot, RT-PCR, immunohistochemistry (IHC), and immunofluorescence (IF) were used to observe the key target of lipid metabolism. In vitro, the final concentration of cholesterol was 100 μmol/L in Caco-cells. Oil red O staining, western blot, RT-PCR and immunofluorescence (IF) were used to observe the changes of lipid metabolism. Finally, the influence of liver X receptor alpha (LXRα) on intestinal cholesterol metabolism was clarified by applying the LXRα inhibitor GSK2033 and siRNA targeting LXRα.

**Results:**

The aortic arch and intestinal villi of the two groups of ApoE^−/−^ mice showed apparent lesions and lipid accumulation, and there were significant changes in a variety of lipids in the plasma and jejunum. Additionally, jejunum LXRα was markedly activated. High cholesterol can significantly activate LXRα in Caco-2 cells. After LXRα was inhibited, the protein level of ATP-binding cassette transporter A1/G5/G8 (ABCA1/G5/G8) decreased, and the quantity and volume of intracellular lipids soared.

**Conclusion:**

In a high-cholesterol environment, the intestine promotes the excretion of cholesterol from the cell through the LXRα-ABCA1/G5/G8 pathway, reduces the intestinal intake of a variety of exogenous cholesterol, and reduces the risk of AS.

**Graphical Abstract:**

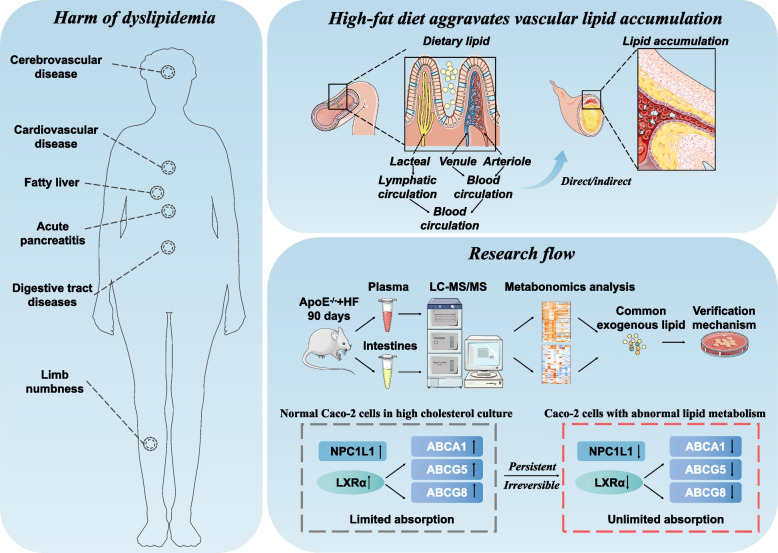

**Supplementary Information:**

The online version contains supplementary material available at 10.1186/s12944-023-01810-6.

## Introduction

A certain correlation between dyslipidemia and atherosclerosis (AS) in clinical [1]. AS is mainly manifested by lipid deposition under the aortic endothelium, abnormal proliferation of smooth muscle, calcification of the middle layer of the artery, and a grand number of necrotic cores [2]. Blood lipids are judged by detecting the levels of four items of blood lipids including total cholesterol (TC), total triglycerides (TG), low-density lipoprotein cholesterol (LDL-c), and high-density lipoprotein cholesterol (HDL-c) to predict the risks of chronic cardiovascular diseases and sudden cardiovascular events [3]. Generally, the lipids in the plasma in the fasting state are mainly derived from the very low-density lipoprotein cholesterol (VLDL-c) endogenously synthesized by the liver [4]. Therefore, drugs that reduce the endogenous synthesis of the liver can reduce the cholesterol ester (CE) and triglyceride (TAG) levels in the plasma [5]. However, in patients with abnormal lipid metabolism such as obesity, diabetes, and hyperlipidemia, intestinal-derived lipids, that is, exogenous lipids, account for a large proportion of plasma lipids [6]. Therefore, reducing the intestinal intake of lipids is considered to be a necessary means to reduce blood lipid levels.

Liver X receptor alpha (LXRα) is considered as a steroid-activated transcription factor [7]. It is mainly expressed in tissues with vigorous cholesterol metabolism and is a key target involved in cholesterol metabolism in macrophages, liver cells, and intestinal cells [8]. Studies have confirmed that activated LXRα can promote cholesterol to return to the liver through reverse transport (RCT) and increase the synthesis of TAG by the liver [9]. However, the expression of intestinal LXRα and its downstream targets under high cholesterol conditions is still unclear. LXRα- ATP-binding cassette transporter A1/G5/G8 (ABCA1/G5/G8) pathway is one of the channels to regulate cellular cholesterol homeostasis in the liver and intestine. When intracellular cholesterol level increases, LXRα, as a nuclear sterol receptor, can encourage intracellular cholesterol transport by up-regulating the expression of ABCA1/G5/G8 [10]. LXRβ can also be activated by low cholesterol levels which ensures the regulation of exogenous cholesterol metabolism in normal cells [7]. Niemann-Pick C1-Like 1 (NPC1L1) is the absorption of exogenous cholesterol and plant sterol, which is opposite to the role played by ABCA1/G5/G8 [11]. The high expression of NPC1L1 reflects the increase in cholesterol uptake in cells. At the same time, in order to clarify the changes in endogenous cholesterol and fatty acids from synthesis, researchers poured attention to the following targets. As an example, acetyl-CoA carboxylase (ACC) acts as a rate-limiting enzyme to regulate fatty acid synthesis. It mainly regulates fatty acid synthesis by catalyzing acetyl coenzyme A to malonyl coenzyme A [12]. What is more, fatty acid synthase (FAS) is the vital enzyme that participates in the conversion from sugar to lipid [13]. Sterol regulatory element binding protein (SREBP) cholesterol regulatory element binding protein mainly exists on the endoplasmic reticulum of cells when intracellular cholesterol is abundant, and its up-regulation and nuclear entry often means that more cholesterol is needed in the cell [14]. In addition, HMG-CoA reductase (HMGCR), a mighty rate-limiting enzyme, can mediate the de novo synthesis of cholesterol. Inhibition of HMGCR is the action mode of statins in lowering plasma cholesterol [15].

In this research, non-targeted lipids metabolomics was used to analyze the plasma and intestinal cholesteryl esters (CE), triglycerides (TAG), phospholipids (PL), and free fatty acids (FFA) of normal C57BL/6 mice and AS mice. This research first identified lipid metabolites that are significantly different in the plasma and intestinal tract. Subsequently, we still confirmed the direct correlation of a variety of exogenous cholesterol to plasma cholesterol. Eventually, these studies found that in high cholesterol conditions, the protein level of LXRα can be up-regulated. Inhibition of LXRα can lead to the decrease of cholesterol efflux mediated by ABCA1/G5/G8 in intestinal cells. In brief, after the excess exogenous cholesterol is absorbed by the intestinal tract, it will be discharged from the cells again through the above channels, and then discharged from the body through the intestinal tract, thus maintaining the relative balance of cholesterol levels.

## Materials and methods

### Mice and treatments

Eight-week-old female C67BL/6 and ApoE^−/−^ mice were purchased from Nanjing Qinglongshan Animal Breeding Center (10 mice in each group). Raised in SPF animal room. Adaptive feeding for a week. The mice in the C57BL/6 + NF group and the ApoE^−/−^ + NF group were fed a normal specific pathogen-free (SPF) diet; the mice in the ApoE^−/−^ + HF group were fed a high-cholesterol SPF diet. (HF, containing 10% fatty oil, 2% cholesterol, 4% whole milk powder, and 0.5%) sodium cholate). All mice were fed for a total of 90 days.

### Caco-2 cell culture and model evaluation

Caco-2 cells were obtained from Fenhui Biotechnology Co., Ltd. (Nanjing, China, CL0060), and were cultured in DMEM high glucose medium (5% CO_2_, 37 °C., 10% fetal bovine serum (FBS), and 1% penicillin/streptomycin). The medium containing cholesterol micelles contains the following components: 100 μM cholesterol, 390 mM oleic acid, 110 μM glyceryl monostearate, and 5 mM taurocholate by autoclaving and ultrasonic treatment at 37℃ for 2 h. Cholesterol needs to be crushed into powder in a mortar in advance. Caco-2 cells were cultured in DMEM high glucose medium containing 5% FBS for 24 h.

### TEM observation of thoracic aorta

The fresh mouse thoracic aorta was washed several times, quickly cut into 1–2 mm^3^ size, immersed in 2.5% glutaraldehyde solution (Wuhan servicebio biology science and technology company, China), and fixed at 4℃ for 2–4 h. Then, different concentrations of ethanol and acetone were used for gradient dehydration, embedding solution infiltration, embedding, and slicing. Finally, it was stained with 2% uranium acetate-lead citrate and observed by transmission electron microscope [16].

### Kit for detecting lipids

Blood sample: Take blood from the ophthalmic vein, the volume ratio of plasma to anticoagulant 4% sodium citrate is 9: 1. Mix it upside down, immediately treat at 4℃ for 10 min at a speed of 3000 rpm, then collect the supernatant. Tissue and intestinal contents: Add 100 μL phosphate-buffered saline (PBS) to every 10 mg sample, grind at 4℃, add the same volume of methanol, shake vigorously, centrifuge at 12000 rpm, then take the supernatant at 4℃ for 10 min; Samples: Add 100 μL PBS to 1 × 10^7^ cells, ground at 4℃, then collect the supernatant, the same volume of methanol is added, shaken vigorously, centrifuged at 12000 rpm for 10 min, then collect the supernatant. The lipid kits include TC, FC, TG, HDL-c, and LDL-c kits (Nanjing Chengjian Biotechnology Research Institute, China) respectively. See the instructions for the specific steps.

### Methodology for non-targeted lipidomics

Sample preparation: 20 μL plasma or jejunum homogenate was placed in a centrifuge tube, then 225 μL ice methanol containing Lyso PE (17:1), SM (17:0), and PE (17:0/17:0) with a concentration of about 5 μg/mL (added lipid as internal standard) was added and vortexed for 10 s, and 750 μL MTBE was added. Vortex for 20 s, then centrifuges at 4℃ and 18000 rpm for 2 min, suck 350 μL of supernatant into a new 1.5 mL centrifuge tube, centrifuge, concentrate and dry for 2 h. At last, prepare a solution with the volume ratio of methanol to toluene of 9: 1, dissolve the sample in 110 μL of the above solution, vortexe and ultrasonicate for 15 min each, and then centrifuge at 18000 rpm for 10 min. Absorb 60 μL supernatant and put it in the injection vial, and examine it in HPLC-Q-TOF/MS system [17].

### Small interfering RNA transfection

Specific small interfering RNAs (siRNAs) against LXRα (sense, 5′-GCTTGCAAACTGGACGATGGAG-3′;antisense, 5′-GACTACGACGGCTGCTACCGT-3′) were synthesized by the Vazyme Company(Nanjing, China). Caco-2 cells (60%-80% confluent monolayer) were seeded in 6-well plates with 2.5 mL of standard mediums. The following day, the cells were transfected with siRNA duplexes (20 nM final concentration) using Lipofectamine™ RNAiMAX reagent (Invitrogen) according to the manufacturer’s instructions. After 72 h, RT-qPCR and western blot analyses were performed to determine the transfection efficiency.

### Statistical analysis

In addition to the data of lipids metabolomics, other data were collected from at least three independent experiments and shown as mean ± SEM (standard error of the mean). One-way ANOVA was used to compare the mean values, and then Student Newman-Keuls (SNK) was post-tested by GraphPad Prism 8.0 software. A *P* value of less than 0.05 was considered statistically significant. In the analysis of lipids metabolomics, the PCA principal components and OPLS-DA partial least squares are obtained through the statistical software (https://www.omicshare.com) and GENE DENOVO Biological Company in Mataboanalysis 3.0 (http://www.metaboanlyst.ca) cloud platform. Discriminant analysis results. Lipid metabolites satisfying *P* value ≤ 0.05 + fold change ≥ 1.5 or ≤ 0.667 are defined as significantly different lipids.

## Results

### High-cholesterol diet-induced AS symptoms and abnormal plasma lipid profile in ApoE^−/−^ mice

**Fig. 1:**
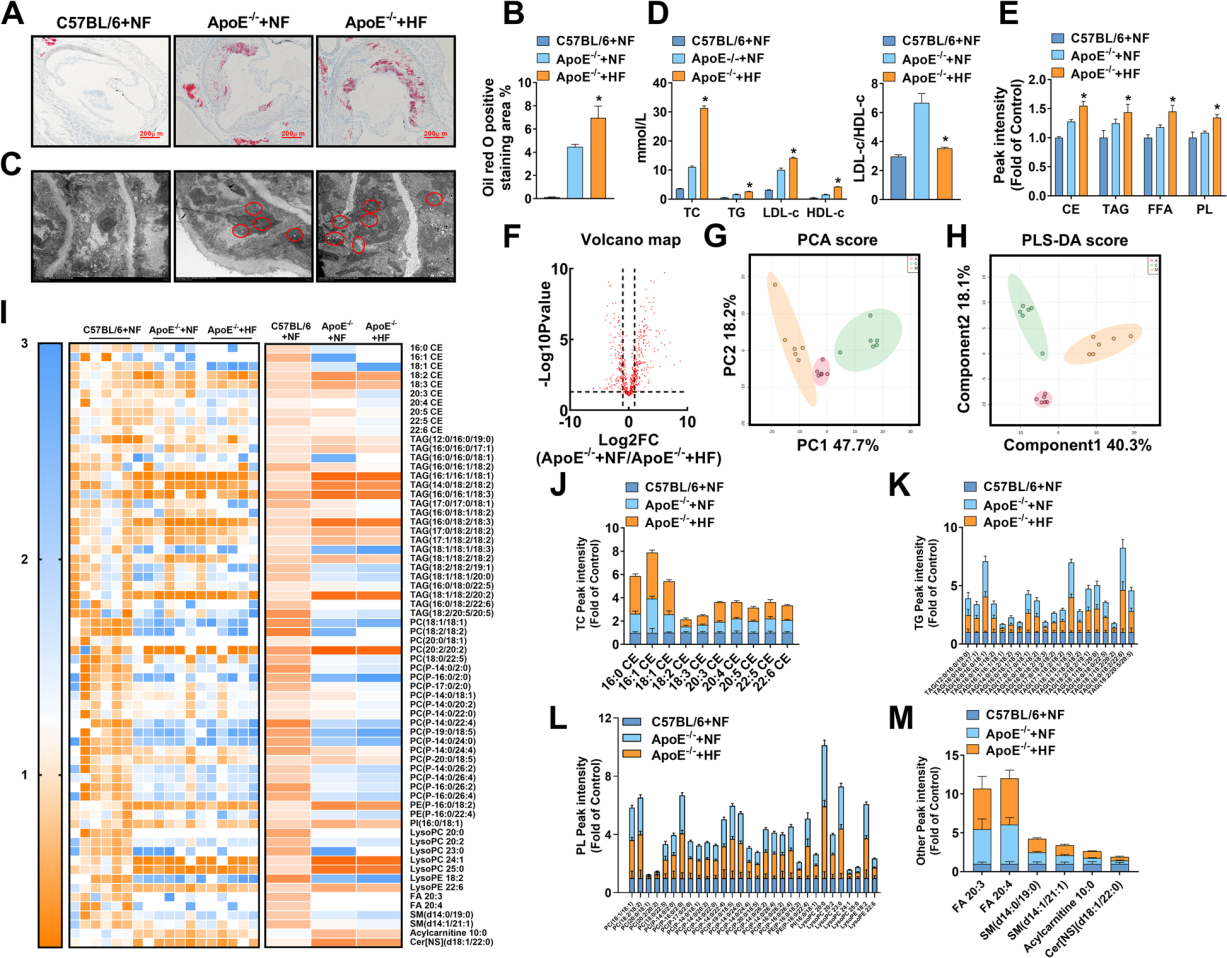
Atherosclerotic lesions and abnormal blood lipid levels of ApoE^−/−^ mice fed a high-cholesterol diet. **A**-**B** Representative images of oil red O staining of the aortic arch and quantitative analysis of the percentage of oil red O positive staining area. Original magnification: 40 × . **C** Representative transmission electron microscope images of subcutaneous lipid droplets in the aortic arch. Original magnification: 1.2 k × . **D** The four levels of blood lipids (left) and the ratio of low-density lipoprotein to high-density lipoprotein (right) by kits. **E** Lipids in plasma samples were extracted and detected by HPLC-Q-TOF/MS. **F** Volcano diagrams of the ApoE^−/−^ + NF group and the ApoE^−/−^ + HF group. **G** PCA score chart, showing the difference between the C57BL/6 + NF group (green), the ApoE^−/−^ + NF group (blue), and the ApoE^−/−^ + HF group (red) in plasma samples. **H** PLS-DA score chart, showing the difference between the C57BL/6 + NF group (green), the ApoE^−/−^ + NF group (blue), and the ApoE^−/−^ + HF group (red) in plasma samples. **I** The heat map showed the difference in plasma lipid levels in each group. **J**-**M** The box plot reflected the relative content of each type of lipid. In all experiments, *n* = 6, the *P* value indicates the comparison with the ApoE^−/−^ + NF group. Values are expressed as mean ± SEM

To clarify that AS appeared in model mice, The aortic arch of three groups of mice was stained with oil red O staining. The results found that the aortic arches of the C57BL/6 mice had no plaque, while the aortic arches of the ApoE^−/−^ + NF group mice and ApoE^−/−^ + HF group mice had obvious lipids deposition (Fig. 1A-B). Next, the researchers observed the presence of lipids to clarify the lipid status of mice in each group, Four items of blood lipids in collected mouse plasma were detected by kits. The results represented that by comparison with the C57BL/6 mice, the levels of four items of blood lipids in two groups of ApoE^−/−^ mice were significantly upgraded, and the HF diet worsened the plasma lipid of ApoE^−/−^ mice (Fig. 1D, left). Since the ratio of LDL-c/HDL-c is positively related to the pressure of cholesterol transportation, the researchers have made statistics on the above indicators of the three groups of mice and found that two ApoE^−/−^ groups mice have significant plasma cholesterol transport loads which were heavier than that of mice in the C57 group (Fig. 1D, right). To explore which specific components of ApoE^−/−^ mice’s blood lipids can be affected by HF, non-targeted lipidomics research has been used to further clarify the lipid categories that produce significant changes. According to the types of lipid metabolites, the results showed that the four main lipid components of CE, TAG, FFA, and PL in the mouse plasma were significantly up-regulated after giving HF diet, (Fig. 1E). The volcano map made of the detected lipid metabolites intuitively shows that there are significant changes in the plasma of the two groups of ApoE^−/−^ mice, and it can be seen that there are more types of lipid metabolites that are significantly up-regulated than down-regulated metabolites (Fig. 1F). To explore the differences in the plasma as a whole, the researchers analyzed the detected plasma metabolites by PCA and PLS-DA. The similarity of lipid metabolites in each group can be expressed by the distance between samples. The farther the distance, the greater the difference between samples, which is reflected in the types and relative abundance of lipids contained. The clustering results of the two mathematical models intuitively show that the HF diet can produce obvious differences in plasma lipid metabolites of ApoE^−/−^ mice (Fig. 1G-H). Finally, the researchers have made statistics on the lipid components detected in the plasma of the mice in this research. The heat map and the box plot showed that compared with the mice in the ApoE^−/−^ + NF group, 10 types of CE, 20 types of TAG, 30 types of PL, 2 types of FFA, 2 types of sphingomyelin (SM), 1 types of acylcarnitine (Acy), and 1 type of ceramide (Cer) were significantly transformed in the plasma of the ApoE^−/−^ + HF group (Fig. 1I-M). These results confirmed that the continuous high-cholesterol diet leads to abnormal blood lipid metabolism, and provided a basis for us to emphasize the intestinal intake of cholesterol.

### High-cholesterol diet-induced jejunum tissue lesions and abnormal jejunum lipid metabolism in ApoE^−/−^ mice

**Fig. 2:**
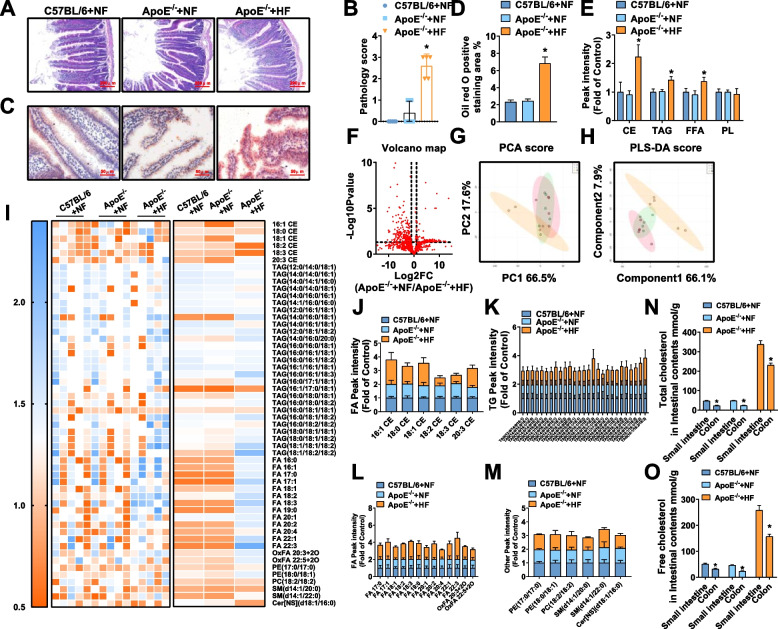
Intestinal lesions and abnormal intestinal lipid levels in ApoE^−/−^ mice fed a high-cholesterol diet. **A**-**B** Representative images of HE staining of the small intestine and evaluation scores of intestinal villi morphology. **C**-**D** Representative images of oil red O staining of proximal jejunum villi and quantitative analysis of the percentage of oil red O positive staining area. Original magnification: 40 × . **E** The lipids of small intestine samples were extracted, detected by HPLC-Q-TOF/MS, and statistically analyzed with different mathematical models. **F** Volcano diagrams of the ApoE^−/−^ + NF group and the ApoE^−/−^ + HF group. **G** PCA score chart, showing the difference between the C57BL/6 + NF group (green), the ApoE^−/−^ + NF group (blue), and the ApoE^−/−^ + HF group (red) in the small intestine samples. **H** The PLS-DA score chart shows the difference between the C57BL/6 + NF group (green), the ApoE^−/−^ + NF group (blue), and the ApoE^−/−^ + HF group (red) in the small intestine samples. **I** The heat map shows the difference in the small intestine lipids in each group. **J**-**M** The box plot reflects the relative content of each type of lipid. **N**–**O** Total cholesterol and free cholesterol in intestinal contents tested by kits. In all experiments, *n* = 5, the *P* value indicates the comparison with the ApoE^−/−^ + NF group. Values are expressed as mean ± SEM

To clarify the influence of a high-cholesterol diet aiming at jejunum lipid intake, the researchers first observed the morphology and structure of the jejunum of the three groups of mice by HE staining. The results showed the length of the jejunum villi of the mice in the ApoE^−/−^ + HF group was significantly shorter than two groups of mice fed the NF diet, swelling, the depth of intestinal crypts increases significantly, showing a higher level of pathology (Fig. 2A-B). Oil red O staining suggested that the volume and number of lipid droplets in jejunum villi in the ApoE^−/−^ + HF group was significantly larger than that in the C57BL/6 + NF group and ApoE^−/−^ + NF group. (Fig. 2C-D). Similar to our study in plasma, to clarify the types of lipids accumulated in the jejunum, the researchers subsequently conducted a non-targeted lipidomics study on the jejunum of the mice in this research. According to the types of lipid metabolites, the total content of the four types of lipid components in the jejunum was counted. The researchers found that the three main lipid components CE, TAG, and FFA in the jejunum of mice in the ApoE^−/−^ + HF group were significantly increased than those in ApoE^−/−^ + NF (Fig. 2E). The volcano map made of the detected lipid metabolites intuitively shows that there are significant changes in the jejunum of the two groups of ApoE^−/−^ mice, and it can be seen that there are more types of lipid metabolites that are significantly up-regulated than down-regulated metabolites (Fig. 2F). PCA and PLS-DA were used to analyze the detected metabolites of jejunum, to explore the differences between groups of jejunum as a whole. The clustering results of the two mathematical models visually suggested the ApoE^−/−^ + NF group and the ApoE^−/−^ + HF group had a clear difference in jejunum lipid metabolites (Fig. 2G-H). Finally, the results of the heat map and the box plot suggested that compared with the ApoE^−/−^ + NF group, 6 types of CE, 27 types of TAG, 15 types of FFA, 3 types of PL, and 2 types of SM in the jejunum of mice in the ApoE^−/−^ + HF group had significant change (Fig. 2I-M). The researchers found that CE containing 16:1, 18:1, and 20:3 ester acyl groups increased synchronously and significantly in both jejunum and plasma. Later, we detected the TC and free cholesterol (FC) content in the contents of the distal colorectal and proximal small intestine of mice by kits, and the ratio can reflect the ability of the small intestine to absorb cholesterol. We found that the absorption fraction of exogenous cholesterol in mice fed the NF diet was about 50%, while that in mice fed the HF diet was about 35% (Fig. 2N-O). Such results suggested that under the condition of a high-cholesterol diet, a large amount of cholesterol is absorbed into the blood by the intestine.

### High-cholesterol diet significantly activated the expression of LXRα in the jejunum of ApoE^−/−^ mice

**Fig. 3:**
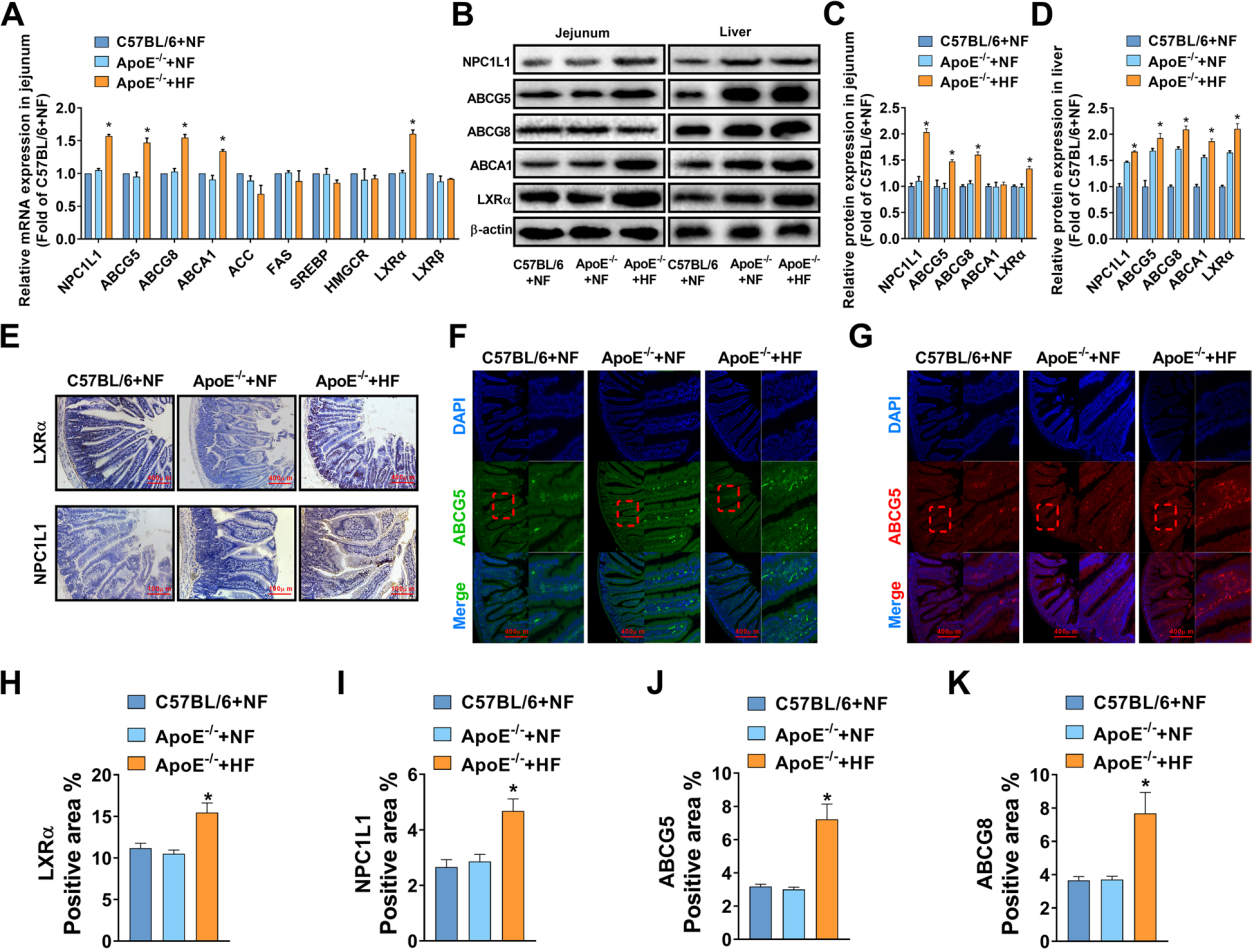
The influence of a high-fat diet on key targets of jejunum lipid metabolism in ApoE^−/−^ mice. **A** The mRNA expression of targets related to lipid metabolism in the jejunum. **B**-**D** Representative western blots and relative quantitative analysis of NPC1L1, ABCG5, ABCG8, and ABCA1 in the proximal jejunum and liver. **E** Representative IHC staining images and quantitative analysis of LXRα and NPC1L1 in the jejunum. **F**-**K** Representative IF images and quantitative analysis of ABCG5 and ABCG8 in the proximal jejunum. In all experiments, *n* = 5, the *P* value indicates the comparison with the ApoE^−/−^ + NF group. Values are expressed as mean ± SEM

The jejunum can not only transport exogenous lipids into the blood in different ways but also can synthesize lipids de novo. To clarify that a high-cholesterol diet can cause the response of key indicators of lipid metabolism in the jejunum, RT-PCR, WB, IHC, and IF were used to study the expression of key lipid metabolism indexes in the jejunum of three groups of mice. The results showed that the high-cholesterol diet can significantly activate the jejunum mRNA levels of NPC1L1, ABCG5, ABCG8, ABCA1, and LXRα in the ApoE^−/−^ + HF mice, but had no statistical change in the ACC, ACS, SREBP1, HMGCR, and LXRβ (Fig. 3A). Then we analyzed the expressions of NPC1L1, ABCG5, ABCG5, ABCA1, and LXRα in the jejunum and liver by WB. We found that a normal diet could not activate the above targets in the jejunum, even if ApoE was knocked out. A high-fat diet can significantly up-regulate the protein expression of the above targets. Different from jejunum, the above targets in the liver were significantly up-regulated when ApoE was knocked out, and a high-fat diet could further up-regulate the protein expression. These results suggested that jejunum lipid metabolism was more biased towards the activation of exogenous lipids, while the liver needs to take into account the metabolism of endogenous lipids and exogenous lipids, and is sensitive to any factors that lead to lipid metabolism. Then the IF, and IHC results showed that compared with the ApoE^−/−^ + NF group, the protein expression of NPC1L1, ABCG5, ABCG8, and LXRα in the ApoE^−/−^ + HF group mice was significantly increased (Fig. 3E-J). These results indicated that a high-cholesterol diet can activate intestinal epithelial cells to excrete exogenous cholesterol mediated by LXRα and ABCG5/G8. Therefore, it can reduce the negative influence of lipid uptake in the jejunum due to the activation of NPC1L1 to a certain degree. However, the regulation mechanism of cholesterol uptake cannot reverse the transport of exogenous cholesterol into the blood by the jejunum.

### The efflux of excessive intracellular cholesterol by Caco-2 cells is mediated by the LXRα-ABCG5/G8 pathway

**Fig. 4:**
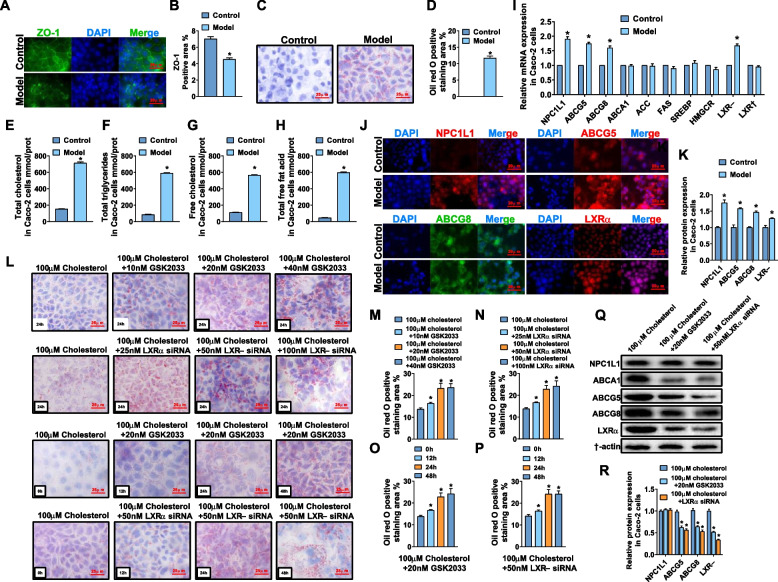
The cholesterol metabolism of intestinal Caco-2 cells is mediated by LXRα. **A**-**B** Representative IF images and quantitative analysis of ZO-1 in Caco-2cells. **C**-**D** Representative images of oil red O staining of Caco-2 cells and quantitative analysis of the percentage of oil red O positive staining area. **E**–**H** Lipid levels in Caco-2 cells tested by kits. **I** The mRNA expression of targets related to lipid metabolism in Caco-2 cells. **J**-**K** Representative IF images and quantitative analysis of NPC1L1, ABCG5, ABCG8, LXRα in Caco-2cells. **L**-**P** representative images of oil red O staining of Caco-2 cells incubated with 100 μmol of cholesterol for 24 h and quantitative analysis of the positive area; **Q**-**R** Representative Western blot and relative quantitative analysis of NPC1L1, ABCA1, ABCG5, ABCG8, and LXRα after incubating Caco-2 cells with 100 μmol of cholesterol for 24 h. In the above experiments, *n* = 5, the *P* value indicates the comparison with the control group. Values are expressed as mean ± SEM

Using Caco-2 cells, the researchers studied the influence of high cholesterol culture on lipid metabolism. The Caco-2 cells were cultured at the bottom of the culture dish, and then the micelles with the final concentration of 100 μM cholesterol were added to the culture medium. By examining the IF results of tight junction protein ZO-1, we found that the protein level of ZO-1 cultured with high cholesterol was significantly lower than that in normal cells (Fig. 4A-B). By observing the size and number of lipid droplets in the oil red O stained image, the researchers found the accumulation of intracellular lipids in Caco-2 cells in the Model group (Fig. 4C-D). Subsequently, through the different lipids test kits, researchers can evaluate the relative content of different types of lipid components in cells. These results suggested that the contents of various lipids in Caco-2 cells increased significantly after the induction of high cholesterol (Fig. 4E-H). To clarify the targets that have significantly changed in Caco-2 cells, the researchers first analyzed the mRNA expression of key lipid metabolism targets by RT-PCR. The above results suggested that the mRNA levels of LXRα, NPC1L1, ABCG5, and ABCG8 were increased significantly (Fig. 4I). The IF results for the above targets were consistent with the RT-PCR results (Fig. 4J-K). Next, the researchers studied the changes in cholesterol metabolism in Caco-2 cells after LXRα was inhibited by giving GSK2033, a specific inhibitor of LXRα and siRNA targeting Continuous application of GSK2033 at 10, 20, and 40 nM or LXRα siRNA at 25, 50, and 100 nM for 24 h can further lead to intracellular lipid accumulation. Moreover, the application of 20 nM GSK2033 or 50 nM LXRα siRNA for 12, 24, and 48 h can lead to the aggravation of intracellular lipid accumulation (Fig. 4L-P). Severe lipid accumulation can be observed in Caco-2 cells by applying 20 nM GSK2033 or 50 nM LXRα siRNA 24 h in advance, the researchers tried to explore the expression of NPC1L1, ABCA1, ABCG5, and ABCG8 when LXRα was inhibited. The researchers found that the protein levels of ABCA1, ABCG5, and ABCG8 were significantly reduced but the expression of NPC1L1 did not change significantly (Fig. 4Q-R).

## Discussion

In this study, the researchers verified the successful preparation of the mouse AS model at the pathological level and detected the levels of four blood lipids. Then, the researchers obtained the lipid profiles of the plasma and jejunum by non-targeted lipidomics analysis. The researchers have clarified the positive correlation between the four types of CE with 16:0, 16:1, 18:1, and 20:3 ester acyl groups that are elevated in plasma and the presence of exogenous lipids uptake by the jejunum. Subsequently, the researchers identified the key targets of intestinal cholesterol metabolism in AS mice and identified the significant influence of the LXRα-ABCG5/G8 pathway on intracellular cholesterol efflux in vitro.

The researchers found that the value of LDL-c/HDL-c in ApoE^−/−^ + HF mice plasma was lower than in ApoE-/- + NF mice, which was a very interesting phenomenon. This result showed that the pressure of ApoE^−/−^ + HF mice on endogenous cholesterol was significantly lower than ApoE^−/−^ + NF mice. Nevertheless, ApoE^−/−^ + HF mice face a higher risk of lipid abnormality than ApoE^−/−^ + NF mice. Our oil red staining results of the aorta in Fig. 1A have confirmed this. The place that can’t be ignored comes from exogenous cholesterol. High-fat feeding leads to a significant increase in the level of exogenous cholesterol absorbed into the blood through the intestine. ApoE^−/−^ + HF mice can absorb excess exogenous cholesterol into the blood circulation through the intestine for a long time, and then transport it to the peripheral tissues for utilization, while the excess exogenous cholesterol that cannot be used in time will be temporarily stored in cells, which will lead to a sharp increase in the cholesterol content of peripheral tissues, so more HDL is needed to transport exogenous cholesterol. Therefore, although the level of HDL-c is compensated, it is still not enough to bear the pressure of reverse cholesterol transport. It should also be noticed that the level of LDL-c is still rising after high-fat feeding, and LDL-c has the potential risk of inducing AS. In a word, although the ratio of LDL-c/HDL-c of ApoE^−/−^ + HF mice decreases, the total cholesterol clearance pressure of ApoE^−/−^ + HF mice is still higher than that of ApoE^−/−^ + NF mice.

Besides, the researchers found that the levels of FA 16:1, FA 18:1, and FA 20:3 in the jejunum of ApoE^−/−^ + HF mice and their corresponding cholesterol esters increased at the same time. These cholesterol esters are the most general type of lipids in vegetable oil which can also find the corresponding lipids in the plasma lipid spectrum. These results indicated that sustained and excessive intake of exogenous lipids, especially cholesterol esters rich in monounsaturated fatty acids, can significantly aggravate dyslipidemia and vascular diseases. In order to reduce AS risk, people need to control dietary lipids, especially oleic acid, and palmitoleic acid. These types of fatty acids are easy to form trans fatty acids at high temperatures which can significantly increase the risk of cardiovascular emergencies. It is an indispensable way to improve dyslipidemia and reduce the risk of CVD by inhibiting intestinal epithelial cells from ingesting exogenous cholesterol or promoting intracellular cholesterol transport to the ileum cavity and reducing the absorption of exogenous cholesterol into the blood. For example, drugs such as cholesterol absorption inhibitor Ezetimibe [18] and bile acid adsorbent Cholestyramine [19] are also used to interfere with the absorption of lipids by intestinal epithelial cells. Using it alone or in combination with other drugs, it can significantly alleviate the clinical symptoms of certain hyperlipidemia patients, while reducing the incidence of cardiovascular and cerebrovascular diseases [20].

Some results suggested that the ApoE gene may not directly participate in the regulation of intestinal lipid metabolism. Different from the plasma lipid profile, no obvious difference was found in the intestinal lipid profile of the C57BL/6 + NF group and ApoE^−/−^NF group, and no obvious pathological changes were found in the intestines of the two groups. However, the knockout of the ApoE gene reduces the uptake of plasma cholesterol by the liver and inhibits the redistribution of de novo cholesterol synthesized by the liver. Unlike in the intestine, ApoE gene knockout significantly changed the expression of LXRα, ABCG5, ABCG8, and NPC1L1 in the liver. We studied the expression of the above proteins in the liver of mice in each group by WB. We found that compared with C57BL/6 + NF mice, the expression of the above proteins in the liver of ApoE^−/−^ + NF mice and ApoE^**−/−**^ + HF mice increased significantly, and the protein expression in the liver of ApoE^−/−^ + HF mice was the highest. The above results suggested that the absorption of cholesterol by the liver will inevitably increase in a high-cholesterol environment. Therefore, under the conditions of ApoE gene knockout and a high-fat diet, the body can reduce the metabolic disorder of liver cholesterol by promoting the liver to excrete unesterified cholesterol into the bile duct or small intestine.

Previous studies have represented that promoting liver cholesterol to produce bile acid is an indispensable way to reduce liver cholesterol levels and improve liver cholesterol metabolism in a short time [21]. However, the intestinal tract reabsorbs bile acids, so it may cause an additional burden on the absorption of cholesterol in the intestinal tract in the long run. In addition, bile acid metabolism is regulated by intestinal flora. Yu Fu’s research further elaborated that the metabolic pathway of liver cholesterol-bile acid can finally regulate liver cholesterol metabolism and alleviate abnormal lipid metabolism by influencing the abundance and species of intestinal flora [22]. In addition, Meng Qing directly explained that different intestinal bacteria, including *Lactobacillus* and *Bifidobacterium*, can alleviate AS in ApoE^−/−^ mice fed with a high-cholesterol diet [23].

Collectively, stimulated by high cholesterol, the small intestine promotes the transfer of cholesterol from intestinal cells to intestinal cavity through LXRα-ABCA1/G5/G8 pathway, which can effectively reduce the absorption of cholesterol in jejunum. Regulating the long-term homeostasis of plasma cholesterol by inhibiting exogenous cholesterol from entering the blood has a positive effect on preventing and improving atherosclerosis caused by dyslipidemia.

## Supplementary Information

**Additional file 1.** Cholesterol micelle suspension. 

**Additional file 2.** Lipids in feed.

**Additional file 3.** Repetition rate. 

**Additional file 4.** Statistics 

**Additional file 5.** Supply materials-materials and methods.

## Data Availability

All the original data and images have been submitted through supplementary materials.

## Abbreviations

ABCG5: ATP binding cassette transporter G5
ABCG8: ATP binding cassette transporter: G8
ABCA1: ATP binding cassette transporter A1
ACC: Acetyl-CoA carboxylase
AS: Atherosclerosis
BSA: Bovine serum albumin
CE: Cholesterol ester
CHD: Coronary heart disease
CM: Chylomicrons
DAG: Diglyceridee
DGAC: Dietary Guidelines Advisory Committee
ECL: Enhanced chemiluminescence
FFA: Free fatty acid
FC: Free cholesterol
FAS: Fatty acid synthase
FBS: Fetal bovine serum
HFD: High-fat diet
HMGCR: Hydroxymethylglutarate monoacyl-CoA reductase
HPLC-Q-TOF/MS: High performance liquid chromatography tandem quadrupole time-of-flight mass spectrometry
LDL-c: Low density lipoprotein cholesterol
LXRα: Liver X receptor α
LXRβ: Liver X receptor β
HDL-c: High density lipoprotein cholesterol
OCT: Optimal cutting temperature compound
PC: Phosphatidylcholine
PE: Phosphatidylethanolamine
PI: Phosphatidylinositol
PBS: Phosphate buffered saline
PBS: Phosphate buffer saline
PMSF: Benzene methylsulfonyl fluoride
PVDF: Polyvinylidene fluoride film
SDS-PAGE: Sodium dodecyl sulfate–polyacrylamide gel electrophoresis
SREBP: Sterol regulatory element binding protein
TAG: Triglyceride
TC: Total cholesterol
TG: Total triglycerides
TBS-T: Tween-20 Tris buffered saline
