# Supplementary material for: Excessive exogenous cholesterol activating intestinal LXRα-ABCA1/G5/G8 signaling pathway can not reverse atherosclerosis in ApoE−/− mice
Source: Lipids Health Dis. 2023 Apr 15;22:51. doi: 10.1186/s12944-023-01810-6 (PMC10105390; doi:10.1186/s12944-023-01810-6)
Supplement: Supplementary file 3 — Additional file 3. Repetition rate. [file 12944_2023_1810_MOESM3_ESM.pdf]

Activated liver X receptor alpha ATP binding cassette transporter G5G8 pathway can alleviate atherosclerosis in ApoE mice

by Yu Xichao

oc (226.78K)
Word count: 5956
Character count: 33720

Activated liver X receptor alpha ATP binding cassette transporter G5G8 pathway can alleviate atherosclerosis in ApoE KD mice

Abstract

Background: The long-term excessive intake of exogenous cholesterol can lead to abnormally elevated blood lipid levels and induce cardiovascular and cerebrovascular diseases. However, the influence and relevance of exogenous cholesterol on plasma cholesterol components are still unclear, and the influence on intestinal lipid metabolism targets needs to be further explored.

Methods: In vivo, the C57BL/6+NF group mice were fed a normal specific pathogen-free (SPF) diet; the ApoE-/-+NF group mice were fed a normal SPF diet; the ApoE-/-+HF group mice were fed a high-cholesterol SPF diet. All mice were fed for a total of 90 days. The plasma and jejunum tissue homogenates were obtained for nontargeted lipid metabolomics and blood lipid testing. The lipid droplets in tissues were observed by transmission electron microscope and oil red O staining. jejunum tissue morphology was observed by HE staining. Western blotting, RT-PCR, immunohistochemistry (IHC), and immunofluorescence (IF) were used to observe the key target of lipid metabolism.. In vitro, the final concentration of cholesterol was 100 μmol/L to culture Caco-2 cells. After oil O staining, observe the number and size of intracellular lipid droplets, and Western blotting, RT-PCR, and immunofluorescence (IF) were used to observe changes in key targets of lipid metabolism. Finally, the infiuence of liver X receptor alpha (LXRα) on intestinal cholesterol metabolism was clarified by applying the LXRα inhibitor GSK2033 and siRNA targeting LXRα.

Results: The aortic arch and intestinal villi of the two groups of ApoE-/- mice showed obvious lesions and lipid accumulation, and there were significant changes in a

variety of lipids in the plasma and jejunum. Additionally, jejunum LXRα was markedly activated. High cholesterol can significantly activate LXRα in Caco-2 cells. After LXRα was inhibited, the protein level of ATP-binding cassette transporter G5/G8 (ABCG5/G8) decreased, and the quantity and volume of intracellular lipids increased significantly.

Conclusion: In a high-cholesterol environment, the intestine promotes the excretion of cholesterol from the cell through the LXRα-ABCG5/G8 pathway, reduces the intestinal intake of a variety of exogenous cholesterol, and reduces the risk of AS.

Keywords: exogenous cholesterol, lipidomics, atherosclerosis, LXRα-ABCG5/G8

1. Introduction

There is a significant positive correlation between dyslipidemia and atherosclerosis (AS)[1]. AS is mainly manifested by lipid deposition under the aortic endothelium, abnormal proliferation of smooth muscle, calcification of the middle layer of the artery, and a large number of necrotic cores[2]. Blood lipids are judged by detecting the levels of four items of blood lipids including total cholesterol (TC), total triglycerides (TG), low-density lipoprotein cholesterol (LDL-c), and high-density lipoprotein cholesterol (HDL-c)in the subjects' plasma to predict the risks of chronic cardiovascular diseases and sudden cardiovascular events[3]. Generally, the lipids in the plasma in the fasting state are mainly derived from the very low-density lipoprotein cholesterol (VLDL-c) endogenously synthesized by the liver[4]. Therefore, drugs that reduce the endogenous synthesis of the liver can infiuenceively reduce the cholesterol ester (CE) and triglyceride (TAG) levels in the plasma[5]. However, in some people with abnormal lipid metabolism such as obesity, diabetes, and hyperlipidemia, intestinal-derived lipids, that is, exogenous lipids, account for a large proportion of plasma lipids[6]. Therefore, reducing the intestinal intake of lipids is

considered to be a necessary means to reduce blood lipid levels.

Liver X receptor alpha (LXRα) is considered as a steroid-activated transcription factor[7]. It is mainly expressed in tissues with vigorous cholesterol metabolism and as a key target involved in cholesterol metabolism in macrophages, liver cells, and intestinal cells[8]. Studies have confirmed that activated LXRα can promote cholesterol to return to the liver through reverse transport (RCT) and increase the synthesis of TAG by the liver[9]. However, the expression of intestinal LXRα and its downstream targets under high cholesterol conditions is still unclear. LXRα-ATP-binding cassette transporter G5/G8 (ABCG5/G8) pathway is one of the channels to regulate cellular cholesterol homeostasis in the liver and intestine[10]. When intracellular cholesterol level increases, LXRα, as a nuclear sterol receptor, can promote intracellular cholesterol transport to extracellular by up-regulating the expression of ABCG5/G8. LXRβ, as a nuclear receptor that can also be activated by low cholesterol levels, ensures the regulation of exogenous cholesterol metabolism in normal cells[11]. It has been proved that the role of cholesterol transporter protein named Niemann-Pick C1-Like 1 (NPC1L1) is the absorption of exogenous cholesterol and plant sterol, which is opposite to the role played by ABCG5/G8[12]. The high expression of NPC1L1 often reflects the increase of cholesterol uptake in cells. At the same time, in order to clarify the changes in endogenous cholesterol and fatty acids from synsynthesis, researchers paid attention to the relevant classical targets. For example, acetyl-CoA carboxylase (ACC) acts as a rate-limiting enzyme to regulate fatty acid synthesis. It mainly regulates fatty acid synthesis by catalyzing acetyl coenzyme A to malonyl coenzyme A[13]. Then, fatty acid synthase (FAS) is the key enzyme participate in the conversion from sugar to lipid[14]. Sterol regulatory element binding protein (SREBP) cholesterol regulatory element binding protein

mainly exists on the endoplasmic reticulum of cells when intracellular cholesterol is abundant, and its up-regulation and nuclear entry often means that more cholesterol is needed in the cell[15]. In addition, HMG-CoA reductase (HMGCR), a powerful rate-limiting enzyme, can mediate the de novo synthesis of cholesterol. Inhibition of HMGCR is the action mode of statins in lowering plasma cholesterol[16].

In this research, nontargeted lipids metabolomics was used to analyze the plasma and intestinal cholesteryl esters (CE), triglycerides (TAG), phospholipids (PL), and free fatty acids (FFA) of normal C57BL/6 mice and AS mice. This research first identified lipid metabolites that are significantly different in the plasma and intestinal tract. Subsequently, we still confirmed the direct correlation of a variety of exogenous cholesterol to plasma cholesterol. Finally, these studies found that in high cholesterol condition, the protein level of LXRα can be up-regulated. Inhibition of LXRα can lead to the decrease of cholesterol efflux mediated by ABCG5/G8 in intestinal cells. In brief, after the excess exogenous cholesterol is absorbed by the intestinal tract, it will be discharged from the cells again through the above channels, and then discharged from the body through the intestinal tract, thus maintaining the relative balance of cholesterol levels.

2. Materials and methods

2.1. Mice and treatments

Eight-week-old female C67BL/6 and ApoE-/- mice were purchased from Nanjing Qinglongshan Animal Breeding Center (n=10). Raised in SPF animal room. The indoor temperature is controlled at 22-26°C, and the humidity is lower than 50%. The light-dark cycle is 12 h, and drinking water and food can be obtained for free. After being fed with normal diet for 1 week, the formal experiment began. The

C57BL/6+NF group mice were fed a normal specific pathogen-free (SPF) diet; the ApoE-/-+NF group mice were fed a normal SPF diet; the ApoE-/-+HF group mice were fed a high-cholesterol SPF diet. (HF, containing 10% fatty oil, 2% cholesterol, 4% whole milk powder, and 0.5%) sodium cholate). At the end of the research, the mice were sacrificed and blood and tissue samples were collected for further evaluation.

2.2. Caco-2 cell culture and model evaluation

Caco-2 cells were obtained from Fenhui Biotechnology Co., Ltd. (Nanjing, China, CL0060), and were prepared in DMEM high glucose medium (5% CO2, 37°C., 10% fetal bovine serum (FBS), and 1% penicillin/streptomycin).The medium containing cholesterol micelles contains the following components: 100 μM cholesterol, 390 mM oleic acid, 110 μM glyceryl monostearate, 5 mM taurocholate by autoclaving and ultrasonic treatment at 37°C for 2 h. Cholesterol needs to be crushed into powder in a mortar in advance. Incubate in DMEM high glucose medium containing 5% FBS for 24 h.

2.3. TEM observation of thoracic aorta

The fresh mouse thoracic aorta was washed several times, quickly cut into 1-2 mm^3 size, immersed in 2.5% glutaraldehyde solution (Wuhan servicebio biology science and technology company, China), and fixed at 4°C for 2-4 h. Then, different concentrations of ethanol and acetone were used for gradient dehydration, embedding solution infiltration, embedding, slicing. Finally, it was stained with 2% uranium acetate-lead citrate and observed by transmission electron microscope[17].

2.4. Kit for detecting lipids

Blood sample: Take blood from the ophthalmic vein, plasma was added at the volume ratio of 9: 1 to ensure that the dosage of 4% sodium citrate anticoagulant in centrifuge tube was enough. mix it upside down, immediately treat at 4°C for 10 min at a speed of 3000 rpm, then collect the supernatant. Tissue: Add 100 μL phosphate-buffered saline (PBS) to every 10mg sample, grind at 4°C, add the same volume of methanol, shake vigorously, centrifuge at 12000 rpm, then take the supernatant at 4°C for 10min; Samples: 1×10^7 cells are added with 100 μL PBS, ground at 4°C, then collect the supernatant, the same volume of methanol is added, shaken vigorously, centrifuged at 12000 rpm for 10 min, then collect the supernatant. The lipid kits include TC, TG, HDL-c, and LDL-c kits (Nanjing Chengjian Biotechnology Research Institute, China) respectively. See the instructions for the specific steps.

2.5. Methodology for nontargeted lipidomics

Sample preparation: 20 μL plasma or jejunum homogenate was placed in a centrifuge tube, then 225 μL ice methanol containing Lyso PE (17:1), SM (17:0) and PE (17:0/17:0) with a concentration of about 5μg/mL (added lipid as internal standard) was added and vortexed for 10 s, and 750 μL MTBE was added. Vortex for 20 s, then centrifuge at 4 C and 18000 rpm for 2 min, suck 350 μL of supernatant into a new 1.5 mL centrifuge tube, centrifuge, concentrate and dry for 2 h. Finally, the sample was dissolved in 110 μL methanol: toluene 9:1 solution, vortexed and ultrasonicated for 15 min each, and then centrifuged at 18000 rpm for 10 min. Suck up 60 μL

supernatant and put it in the injection vial, and analyze it in HPLC-Q-TOF/MS system[18].

2.6 Small interfering RNA transfection

Specific small interfering RNAs (siRNAs) against LXRα (sense, 5′-GCTTGCAAACTGGACGATGGAG-3′; antisense, 5′-GACTACGACGGCTGCTACCGT-3′) were synthesized by the Vazyme Company(Nanjing, China). Caco-2 cells (60%-80% confluent monolayer) were seeded in 6-well plates with 2.5 ml of standard medium. The following day, the cells were transfected with siRNA duplexes (20 nM final concentration) using Lipofectamine™ RNAiMAX reagent (Invitrogen) according to the manufacturer's instructions. After 72 h, RT-qPCR and western blot analyses were performed to determine transfection efficiency.

3. Results

3.1. High-cholesterol diet-induced AS symptoms and abnormal plasma lipid profile in ApoE-/- mice.

In order to clarify that AS appeared in model mice, The aortic arch of three groups of mice was stained with oil red O. The results found that the aortic arches of the C57BL/6 mice had no plaques, while the aortic arches of the ApoE-/-+NF group mice and ApoE-/-+HF group mice had obvious lipids deposition (Figure 1A-B). Next, the researchers observed the presence of lipids to clarify the lipid status of mice in the each groups, Four items of blood lipids in collected mouse plasma were detected by kits. The results showed that compared with the C57BL/6 mice, the levels of four items of blood lipids in two groups of ApoE-/- mice were significantly upgraded, and

the HF diet worsened the plasma lipid of ApoE-/- mice (Figure 1D, left). Since the ratio of LDL-c/HDL-c is positively related to the pressure of cholesterol transportation., the researchers have made statistics on the above indicators of the three groups of mice, and found that two ApoE-/- groups mice have significant plasma cholesterol transport load which were heavier than that of mice in the C57 group (Figure 1D, right). In order to explore which specific components of ApoE-/- mice's blood lipids can be affected by HF, nontargeted lipidomics research has been used to further clarify the lipid categories that produce significant changes. According to the types of lipid metabolites, the results showed that four main lipid components of CE, TAG, FFA, and PL in the mouse plasma were significantly up-regulated after giving HF diet, (Figure 1E). The volcano map made of the detected lipid metabolites intuitively shows that there are significant changes in the plasma of the two groups of ApoE-/- mice, and it can be seen that there are more types of lipid metabolites that are significantly up-regulated than downregulated metabolites (Figure 1F). In order to explore the differences in the plasma as a whole, the researchers analyzed the detected plasma metabolites by PCA and PLS-DA. The clustering results of the two mathematical models intuitively show that HF diet can produce obvious differences in plasma lipid metabolites of ApoE-/- mice (Figure 1G-H). Finally, the researchers have made statistics on the lipid components detected in the plasma of the mice in this research. The heat map and box plot showed that compared with the mice in ApoE-/-+NF group, 10 types of CE, 20 types of TAG, 30 types of PL, 2 types of FFA, 2 types of sphingomyelins (SM), 1 types of acylcarnitine (Acy), and 1 types of ceramide (Cer) were significantly changed in the plasma of the ApoE-/-+HF group (Figure 1I-M). These results confirmed that the continuous high-cholesterol diet leads to abnormal blood lipid metabolism, and provided a basis for us to focus on the

intestinal intake of cholesterol.

3.2. High-cholesterol diet-induced jejunum tissue lesions and abnormal jejunum lipid metabolism in ApoE-/- mice.

In order to clarify the infiuence of a high-cholesterol diet aiming at jejunum lipid intake, the researchers first observed the morphology and structure of the jejunum of the three groups of mice by HE staining. The results showed the length of the jejunum villi of the mice in the ApoE-/-+HF group were significantly shorter then two groups of mice fed NF diet, Swelling, the depth of intestinal crypts increases significantly, showing a higher level of pathology (Figure 2A-B). Oil red O staining suggested that the volume and number of lipid droplets in jejunum villi in the ApoE-/-+HF group was significantly larger than that in the C57BL/6+NF group and ApoE-/-+NF group. (Figure 2C-D). Similar to our study in plasma, to clarify the types of lipids accumulated in the jejunum, the researchers subsequently conducted a nontargeted lipidomics study on the jejunum of the mice in this research. According to the types of lipid metabolites, the total content of the four types of lipid components in the jejunum of the each groups of mice were counted. The researchers found that the three main lipid components CE, TAG, and FFA in the jejunum of mice in the ApoE-/-+HF group were significantly increased than those in ApoE-/-+NF (Figure 2E). The volcano map made of the detected lipid metabolites intuitively shows that there are significant changes in the jejunum of the two groups of ApoE-/- mice, and it can be seen that there are more types of lipid metabolites that are significantly up-regulated than downregulated metabolites (Figure 2F). PCA and PLS-DA were used to analyze the detected metabolites of jejunum, so as to explore the differences between groups of jejunum as a whole. The clustering results of the two mathematical models visually suggested the ApoE-/-+NF group and the ApoE-/-+HF group had a clear difference in

jejunum lipid metabolites (Figure 2G-H). Finally, the results of the heat map and box plot suggested that compared with the ApoE-/-+NF group, 6 types of CE, 27 types of TAG, 15 types of FFA, 3 types of PL, and 2 types of SM in the jejunum of mice in the ApoE-/-+HF group had significantcchange (Figure 2I-M). Later, the researchers found that CE containing 16:1, 18:1, and 20:3 ester acyl groups increased synchronously and significantly in both jejunum and plasma. Such results suggested that under the condition of a high-cholesterol diet, the cholesterol in the circulating blood is partly derived from the intestinal intake of cholesterol.

3.3. High-cholesterol diet significantly activated the expression of LXRα in the jejunum of ApoE-/- mice.

The jejunum can not only transport exogenous lipids into the blood in different ways but also can synthesize lipids de novo. In order to clarify that high-cholesterol diet can cause the response of key indicators of lipid metabolism in jejunum, RT-PCR, WB, IHC, and IF were used to study the expression of key lipid metabolism indexes in the jejunum of two groups of mice. The results showed that compared with the ApoE-/-+NF group of mice, the high-cholesterol diet can significantly activate the jejunum mRNA levels of NPC1L1, ABCG5, ABCG8, ABCA1, and LXRα in the ApoE-/-+HF mice, but had no statistical change in the ACC, ACS, SREBP1, HMGCR, and LXRβ (Figure 3B-C). Then the WB, IF, and IHC results showed that compared with the ApoE-/-+NF group, the protein expression of NPC1L1, ABCG5, ABCG8, and LXRα in the ApoE-/-+HF group mice was significantly increased (Figure 3D-K). These results indicated that a high-cholesterol diet can activate intestinal epithelial cells to excrete exogenous cholesterol mediated by LXRα and ABCG5/G8. Therefore, it can reduce the negative infiuence of lipid uptake in the jejunum due to the activation of NPC1L1 to a certain degree. However, the negative regulation

mechanism of cholesterol uptake cannot reverse the transport of exogenous cholesterol into the blood by the jejunum.

3.4. The efflux of excessive intracellular cholesterol by Caco-2 cells is mediated by the LXRα-ABCG5/G8 pathway.

Using Caco-2 cells, the researchers studied the infiuence of high cholesterol culture on the lipid metabolism.. The Caco-2 cells were cultured at the bottom of the culture dish, and then the micelles with the final concentration of 100μM cholesterol were added to the culture medium. By analyzing the IF results of tight junction protein ZO-1, we found that the protein level of ZO-1 cultured with high cholesterol was significantly lower than that in normal cells (Figure 4A-B). By observing the size and number of lipid droplets in the oil red O stained image, the researchers found the accumulation of intracellular lipids in Caco-2 cells in the Model group (Figure 4C-D). Subsequently, through the different lipids test kits, researchers can evaluate the relative content of different types of lipid components in cells.These results suggested that the contents of various lipids in Caco-2 cells increased significantly after the induction of high cholesterol (Figure 4E-H). In order to clarify the targets that have significantly changed in Caco-2 cells, the researchers first analyzed the mRNA expression of key lipid metabolism targets by RT-PCR. Above results suggested that the mRNA levels of LXRα, NPC1L1, ABCG5, and ABCG8 were increased significantly (Figure 4I). The IF results for the above targets were consistent with the RT-PCR results (Figure 4J-K). Next, the researchers studied the changes of cholesterol metabolism in Caco-2 cells after LXRα was inhibited by giving GSK2033, a specific inhibitor of LXRα and siRNA targeting LXRα respectively. Then under the above conditions, oil Red O indicated that the contents of various lipids in Caco-2 cells increased significantly after applying high cholesterol (Figure 4L-M).

Subsequently,the researchers found that after inhibiting LXRα, the protein levels of ABCG5 and ABCG8 was significantly reduced (Figure 4N-O).

4. Discussion

This research is directly based on the intake of cholesterol in the intestine. Through the analysis of plasma and intestinal lipidomics, direct evidence of AS caused by exogenous cholesterol is found, and the specific cholesterol categories are given. Then, the role and mechanism of the intestinal tract in regulating plasma cholesterol levels were clarified. In this study, the researchers verified the successful preparation of the mouse AS model at the pathological level and detected the levels of four blood lipids. Then, the researchers obtained the lipid profiles of the plasma and jejunum by nontargeted lipidomics analysis. Through the comparison, the researchers have clarified the positive correlation between the four types of CE with 16:0, 16:1, 18:1, and 20:3 ester acyl groups that are elevated in plasma and the presence of exogenous lipids uptake by the jejunum. Although it was difficult to conclude that the four types of CE detected in plasma must originate from the uptake of the jejunum, the researchers found that the levels of FA 16:1, FA 18:1, and FA 20:3 in the jejunum of mice in ApoE-/-+HF group were also coincidental increased. It is the most common type of fatty acid in food and can be used as a precursor for the synthesis of cholesterol esters in the jejunum. In addition, FFA 20:3, as an important member of essential fatty acids, generally depends on obtaining from food, especially plant seed oil. Different from the plasma lipid profile, no obvious difference was found in the intestinal lipid profile of C57BL/6+NF group and ApoE-/-NF group, and no obvious pathological changes were found in the intestines of the two groups. These results suggested that ApoE gene may not directly participate in the regulation of intestinal lipid metabolism. Compared with the mice in ApoE-/-+NF group, the intestinal lipids

in ApoE-/-+HF group, including TC, TG and FA, all increased significantly, among which the relative contents of 16: 0, 18: 0, 18: 1 and 20: 3 cholesterol esters increased significantly. And the content of various monounsaturated fatty acids, including 18: 1 fatty acids, was also significantly increased. These results indicate that sustained and excessive intake of exogenous lipids, especially cholesterol esters rich in monounsaturated fatty acids, can significantly aggravate dyslipidemia and vascular diseases.Although the plasma lipid metabolism of ApoE-/-+NF mice was significantly abnormal, the intestinal lipid metabolism of ApoE-/+NF mice did not change significantly compared with C56BL/6 mice. This is due to the knockout of the ApoE gene sharply reduced the ability of the mouse liver to metabolize plasma cholesterol, so a large number of exogenous lipids could not be normally taken up by the liver and stayed in the blood. Therefore, the researchers speculated that the four types of CE derived from food played a negative role in the process of AS in mice. The above results provided an important basis for us to study the metabolism of exogenous cholesterol in the intestine. Subsequently, the researchers identified the key targets of intestinal cholesterol metabolism in AS mice and identified the significant influence of the LXRα-ABCG5/G8 pathway on intracellular cholesterol efflux in vitro

The small intestine is an important place for the absorption of nutrients such as lipids, monosaccharides, and proteins[19]. Most of the lipids are absorbed by the upper 2/3 segment of the jejunum[20]. The process of most lipids being transported into the blood in the intestine requires emulsification and hydrolysis in the intestinal lumen, Then lipoprotein particles are synthesized in intestinal cells, and finally transported to the body through lymphatic capillaries in intestinal villi[21]. Finally, in the form of chylomicrons (CM), they are transported to the blood through the lymphatic circulation[22]. Under the action of lipoprotein lipase (LPL) in the blood, CM

hydrolyzes free fatty acids (FFA) and is taken up by peripheral tissue cells, and the remaining CM residues are taken up by low-density lipid receptor-related protein (LRP) on the surface of liver cells[23]. Therefore, after eating, there will be a significant increase in blood lipid levels which may take a negative influence on the body[24]. A considerable number of pre-clinical and clinical research have found that it is important to control the intake of exogenous lipids to reduce the risk of metabolic diseases[25]. On the one hand, exogenous lipids transported back to the liver can be used as raw materials for endogenous lipid synthesis[26]. On the other hand, a large number of studies have confirmed that long-term excessive intake of foods rich in lipids, especially cholesterol, will increase the burden of liver metabolism, destroy the homeostasis of liver synthesis and metabolism of lipids, and ultimately endanger the liver, heart, brain, and peripherals organization[27]. Therefore, it is an indispensable way to improve dyslipidemia and reduce the risk of CVD by inhibiting intestinal epithelial cells from ingesting exogenous cholesterol or promoting intracellular cholesterol transport to the ileum cavity and reducing the absorption of exogenous cholesterol into the blood. For example, drugs such as cholesterol absorption inhibitor Ezetimibe[28] and bile acid adsorbent Cholestyramine[29] are also used to interfere with the absorption of lipids by intestinal epithelial cells. Using it alone or in combination with other drugs, it can significantly alleviate the clinical symptoms of certain hyperlipidemia patients, while reducing the incidence of cardiovascular and cerebrovascular diseases[30].

For a long time, four items of blood lipid tests have been a reliable method to judge blood lipid status[31]. Cholesterol, as a skeleton component of cell structure, maintains cell stability, but the excessively high cholesterol content in the blood is an independent risk factor for coronary heart disease (CHD)[32]. As an indirect energy

substance, triglycerides have been controversial as to whether it directly induces the occurrence of cardiovascular diseases (CVD)[33]. A recent study has confirmed that triglyceride-rich lipoprotein cholesterol, small dense LDL-c is also an independent risk factor affecting CVD[34]. LDL-c is a product of very low-density lipoprotein (VLDL) metabolism, and it has the characteristics of being easily oxidized and acetylated[35]. So that it becomes a risk factor leading to AS. Therefore, LDL-c is also an important indicator for the pharmacodynamic evaluation of lipid-lowering drugs[36]. The function of HDL-c is mainly to transport excess unused cholesterol from peripheral tissues. Free cholesterol discharged from peripheral tissues is esterified by lysolecithin phosphatidyltransferase (LCAT) and then loaded by HDL-c, reducing the deposition of cholesterol in peripheral tissues[37]. Then the cholesteryl ester transfer protein (CETP) transfers CE to low-density lipoprotein (LDL), which is finally taken up by the low-density lipoprotein cholesterol receptor (LDLR) on the surface of liver cells[38]. This shows that HDL-c plays an important role in cholesterol efflux, and plays an anti-AS infiuence by promoting the reverse cholesterol transport (RCT) process. In clinical studies, increasing HDL conversion rate by hydrolyzing HDL phospholipids and driving HDL particle clearance has been proved to be an effective method to reduce CVD risk[39]. In brief, the synthesis, transport, and metabolism of lipids in the body involve complex regulatory mechanisms and the four blood lipids cannot reflect the differences in specific lipid components. Therefore, it is impossible to study the four blood lipids to clarify the specific lipid metabolism mechanism at the cell level. Therefore, the researchers tried to explore the changes in different types of lipid components in plasma through nontargeted lipid metabolomics, and initially established the relationship between jejunum lipid components and plasma lipid components by using cholesterol as an

entry point.

To explore the infiuence of exogenous cholesterol aim to the targets and pathways of jejunal cholesterol metabolism, the researchers chose to use Caco-2 cells with high cholesterol conditions to imitate the transport of cholesterol by small intestine. Caco-2 cells have many characteristics similar to jejunal absorption cells, such as microvilli structure, hydrolase, and carrier transport system, and the cells are in a tightly connected state[40]. Therefore, the Caco-2 cell monolayer model is widely used in the study of the absorption and transport mechanism of nutrients and therapeutic drugs including sugars, lipids, proteins, vitamins, water, and inorganic salts[41]. In this study, Caco-2 cells were cultured until a tightly connected monolayer was formed between the cells. As excessive lipid levels may damage cells, it is necessary to adjust the cholesterol content in the culture medium so that cells can survive in lipid culture conditions and have the ability to transport lipids. By the way, the researchers explored the mechanism of lipid metabolism in the jejunum under high cholesterol culture conditions, and clarified the positive role of the LXRα-ABCG5/G8 pathway in the jejunum, providing new research directions for the prevention and treatment of hypercholesterolemia.

It is worth mentioning that the American Dietary Guidelines Advisory Committee (DGAC) issued a new dietary recommendation: no longer restrict residents' cholesterol intake[42]. However, this does not mean that exogenous cholesterol will not affect plasma cholesterol homeostasis. When people state the fact that exogenous cholesterol is harmless, they often supplementally explain that the increased de novo cholesterol synthesized by the liver can supplementally maintain the homeostasis of plasma cholesterol after inhibiting the absorption pathway. Unfortunately, in clinical research cases, patients with abnormal plasma cholesterol have liver steatosis, glucose,

lipid metabolism disorders, and other diseases that account for a large proportion[43]. The lipid metabolism capacity of the above population is no longer sufficient to maintain the balance between source and exogenous cholesterol. The intestine is an important place for cholesterol absorption, so the combination of drugs for improving liver cholesterol metabolism and drugs for inhibiting intestinal cholesterol absorption in clinical treatment plans has achieved the purpose of regulating patients' abnormal plasma cholesterol levels.

For a long time, pharmacologists have explored the mechanism of de novo synthesis of cholesterol by the liver, and then developed a variety of drugs to improve dyslipidemia, which brought good news to patients with dyslipidemia[44]. In addition, as an organ for recycling cholesterol, the injured liver inhibits the process of reassembling cholesterol taken from the intestines into lipoprotein granules that supply the whole body cells, which leads to a high level of cholesterol in the circulating blood for a long time[45]. Therefore, studying the metabolic pathway and mechanism of the liver under cholesterol pressure will be more helpful to study the lipid metabolism process in a pathological state. Previous studies have shown that promoting liver cholesterol to produce bile acid is an indispensable way to reduce liver cholesterol level and improve liver cholesterol metabolism in a short time[46]. However, the intestinal tract reabsorbs bile acids, so it may cause an additional burden on the absorption of cholesterol in the intestinal tract in the long run. In addition, because bile acid metabolism is regulated by intestinal flora, many researchers gradually focus on the intestinal tract. Yu Fu's research further elaborated that the metabolic pathway of liver cholesterol-bile acid can finally regulate liver cholesterol metabolism and alleviate abnormal lipid metabolism by influencing the abundance and species of intestinal flora.[47] In addition, Meng et al. directly explained that

different intestinal bacteria, including *Lactobacillus* and *Bifidobacterium*, can alleviate AS in ApoE-/- mice fed with high-cholesterol diet[48].

In summary, through the above research, can draw the following conclusions: from the perspective of the cholesterol metabolism mechanism, under the induction of high cholesterol in the outside world, jejunum LXRα is significantly activated, which promotes the transportation of cholesterol return to the intestinal lumen through ABCG5/G8, and reduces the absorption of cholesterol in the jejunum. Regulating plasma cholesterol homeostasis in this way has a positive infiuence on preventing and improving AS induced by dyslipidemia. In clinical treatment, patients need to control dietary lipids, especially lipids rich in oleic acid and palmitoleic acid, which are rich in monounsaturated fatty acids. These lipids are often abundant in vegetable oil and butter. On the one hand, these fatty acids are easy to form trans fatty acids at high temperatures. On the other hand, it can synthesize cholesterol ester in the intestinal tract and enter the blood through lymphatic circulation, increasing the risk of cardiovascular and cerebrovascular diseases. In addition, it is necessary to reduce the use of egg yolk and animal internal organs, which can properly supplement the intake of fish and beef.

Figure legends

Figure 1. Atherosclerotic lesions and abnormal blood lipid levels of ApoE-/- mice fed a high-cholesterol diet.

8-week-old female C57BL/6 mice and ApoE-/- mice were given a normal diet for 12 weeks, respectively, and a 8-week-old female ApoE-/- mice were given a high-cholesterol diet for 12 weeks. (A-B) Representative images of oil red O staining of the aortic arch and quantitative analysis of the percentage of oil red O positive

staining area. Original magnification: 40×. (C) Representative transmission electron microscope images of subcutaneous lipid droplets in the aortic arch. Original magnification: 1.2k×. (D) The four levels of blood lipids (left) and the ratio of low-density lipoprotein to high-density lipoprotein (right) by kits. (E) The lipids of plasma samples were extracted, detected by HPLC-Q-TOF/MS, and statistically analyzed according to different mathematical models. (F) Volcano diagrams of the ApoE-/-+NF group and the ApoE-/-+HF group. (G) PCA score chart, showing the difference between the C57BL/6+NF group (green), the ApoE-/-+NF group (blue), and the ApoE-/-+HF group (red) in plasma samples. (H) PLS-DA score chart, showing the difference between the C57BL/6+NF group (green), the ApoE-/-+NF group (blue), and the ApoE-/-+HF group (red) in plasma samples. (I) The heat map shows the difference in lipid levels between the each groups in plasma under given conditions. (J-M) The box plot reflects the relative content of each type of lipid. In all experiments, n=6, the *P* value indicates the comparison with the ApoE-/-+NF group. Values are expressed as mean ± SEM.

Figure 2. Intestinal lesions and abnormal intestinal lipid levels in ApoE-/- mice fed a high-cholesterol diet.

(A-B) Representative images of HE staining of the small intestine and evaluation scores of intestinal villi morphology. (C-D) Representative images of oil red O staining of proximal jejunum villi and quantitative analysis of the percentage of oil red O positive staining area. original magnification: 40×. (E) The lipids of small intestine samples were extracted, detected by HPLC-Q-TOF/MS, and statistically

analyzed according to different mathematical models. (F) Volcano diagrams of the ApoE-/-+NF group and the ApoE-/-+HF group. (G) PCA score chart, showing the difference between the C57BL/6+NF group (green), the ApoE-/-+NF group (blue), and the ApoE-/-+HF group (red) in the small intestine samples. (H) The PLS-DA score chart shows the difference between the C57BL/6+NF group (green), the ApoE-/-+NF group (blue), and the ApoE-/-+HF group (red) in the small intestine samples. (I) The heat map shows the difference in lipid metabolites in the small intestine between the each groups under given conditions. (J-M) The box plot reflects the relative content of each type of lipid. In all experiments, n=5, the *P* value indicates the comparison with the ApoE-/-+NF group. Values are expressed as mean ± SEM.

Figure 3. The infiuence of a high-fat diet on key targets of jejunum lipid metabolism in ApoE-/- mice.

The mRNA expression of targets related to lipid metabolism in the jejunum. (B-C) Representative western blots and relative quantitative analysis of NPC1L1, ABCG5, ABCG8, and ABCA1 in the proximal jejunum (D-G) Representative IHC staining images and quantitative analysis of LXRα and NPC1L1 in the jejunum. (H-K) Representative IF images and quantitative analysis of ABCG5 and ABCG8 in the proximal jejunum. In all experiments, n=5, the *P* value indicates the comparison with the ApoE-/-+NF group. Values are expressed as mean ± SEM.

Figure 4. The cholesterol metabolism of intestinal Caco-2 cells is mediated by LXRα.

(A-B) Representative IF images and quantitative analysis of ZO-1 in Caco-2cells.

(C-D) Representative images of oil red O staining of Caco-2 cells and quantitative analysis of the percentage of oil red O positive staining area. (E-H) Lipid levels in Caco-2 cells by kits. (I) The mRNA expression of targets related to lipid metabolism in Caco-2 cells. (J-K) Representative IF images and quantitative analysis of NPC1L1, ABCG5, ABCG8, LXRα in Caco-2cells. (L-M) representative images of oil red O staining of Caco-2 cells incubated with 100 μmol of cholesterol for 24 h and quantitative analysis of the positive area; (N-O) Representative Western blot and relative quantitative analysis of NPC1L1, ABCG5, ABCG8, and LXRα after incubating Caco-2 cells with 100 μmol of cholesterol for 24 h. In the above experiments, n=5, the *P* value indicates the comparison with the control group. Values are expressed as mean ± SEM.

Abbreviation

ABCG5: ATP binding cassette transporter G5; ABCG8: ATP binding cassette transporter: G8; ABCA1: ATP binding cassette transporter A1; ACC: acetyl-CoA carboxylase; AS: atherosclerosis; BSA: bovine serum albumin; CE: cholesterol ester; CHD: coronary heart disease; CM: chylomicrons; DAG: diglyceridee; DGAC: Dietary Guidelines Advisory Committee; ECL: enhanced chemiluminescence; FFA: free fatty acid; FC: free cholesterol; FAS: Fatty acid synthase; FBS: fetal bovine serum; HFD: high-fat diet; HMGCR: hydroxymethylglutarate monoacyl-CoA reductase; HPLC-Q-TOF/MS: High performance liquid chromatography tandem quadrupole time-of-flight mass spectrometry; LDL-c: low density lipoprotein cholesterol; LXRα: liver X receptor α; LXRβ: liver X receptor β;HDL-c:high density

lipoprotein cholesterol; OCT: optimal cutting temperature compound; PC: phosphatidylcholine; PE: phosphatidylethanolamine; PI: phosphatidylinositol; PBS: phosphate buffered saline; PBS: phosphate buffer saline; PMSF: benzene methylsulfonyl fluoride; PVDF: polyvinylidene fluoride film; SDS-PAGE: sodium dodecyl sulfate-polyacrylamide gel electrophoresis; SREBP: Sterol regulatory element binding protein; TAG: triglyceride; TC: total cholesterol; TG: total triglycerides; TBS-T: Tween-20 Tris buffered saline;

Activated liver X receptor alpha ATP binding cassette transporter G5G8 pathway can alleviate atherosclerosis in ApoE mice

ORIGINALITY REPORT

13%	11%	11%	1%
SIMILARITY INDEX	INTERNET SOURCES	PUBLICATIONS	STUDENT PAPERS

PRIMARY SOURCES

1	static-site-aging-prod2.impactaging.com<br>Internet Source	5%
2	www.ncbi.nlm.nih.gov<br>Internet Source	1%
3	worldwidescience.org<br>Internet Source	1%
4	www.science.gov<br>Internet Source	1%
5	Xi-Chao Yu, Yu Fu, Yun-Hui Bi, Wei-Wei Zhang et al. "Alisol B 23-acetate activates ABCG5/G8 in the jejunum via the LXRα/ACAT2 pathway to relieve atherosclerosis in ovariectomized $ApoE^{-/-}$ mice", Aging, 2020<br>Publication	1%
6	Yu Fu, Han Fen, Xue Ding, Qing-Hai Meng et al. "Alisol B 23-acetate adjusts bile acid metabolisim via hepatic FXR-BSEP signaling	1%

activation to alleviate atherosclerosis", Phytomedicine, 2022
Publication

7 Submitted to Unizin, LLC
Student Paper
<1 %

8 portlandpress.com
Internet Source
<1 %

9 X.J. LENG, X.F. WU, J. TIAN, X.Q. LI, L. GUAN, D.C. WENG. "Molecular cloning of fatty acid synthase from grass carp (Ctenopharyngodon idella) and the regulation of its expression by dietary fat level", Aquaculture Nutrition, 2012
Publication
<1 %

10 theses.gla.ac.uk
Internet Source
<1 %

11 Lei Guo, Zhizhen Lai, Yanmin Wang, Zhili Li. " probing changes in fatty-acyl chain length and desaturation of lipids in cancerous areas using mass spectrometry imaging ", Journal of Mass Spectrometry, 2020
Publication
<1 %

12 www.mdpi.com
Internet Source
<1 %

13 Xiaoling Li, Mengjiao Yang, Hang Sun, Md Reyad ul Ferdous, Ling Gao, Jiajun Zhao, Yongfeng Song. "Liver cyclophilin D deficiency inhibits the progression of early NASH by
<1 %

ameliorating steatosis and inflammation", Biochemical and Biophysical Research Communications, 2022
Publication

14	www.frontiersin.org<br>Internet Source	<1 %
15	open.library.ubc.ca<br>Internet Source	<1 %
16	www.spandidos-publications.com<br>Internet Source	<1 %
17	Peng Wu, Zhengquan Huang, Jinjun Shan, Zichen Luo et al. "Interventional effects of the direct application of “Sanse powder” on knee osteoarthritis in rats as determined from lipidomics via UPLC-Q-Exactive Orbitrap MS", Chinese Medicine, 2020<br>Publication	<1 %
18	aging-us.com<br>Internet Source	<1 %
19	lipidworld.biomedcentral.com<br>Internet Source	<1 %
20	slideplayer.com<br>Internet Source	<1 %
21	www.oncotarget.com<br>Internet Source	<1 %

22	Eleni A. Karavia. "Deficiency in apolipoproteinE has a protective effect on diet-induced nonalcoholic fatty liver disease in mice : ApolipoproteinE and diet-induced NAFLD", FEBS Journal, 09/2011<br>Publication	<1 %
23	Yinmei Liang, Ling Sun, Feng Rong, Xingxing Han, Xinai Ma, Xiaomin Deng, Mengdi Cheng, Jinjun Shan, Wei Li, Tingming Fu. "Inhalation of tetrandrine liposomes for the treatment of bleomycin induced idiopathic pulmonary fibrosis", Journal of Drug Delivery Science and Technology, 2022<br>Publication	<1 %
24	highcholesterolcure.info<br>Internet Source	<1 %
25	pace-cme.org<br>Internet Source	<1 %
26	www.coursehero.com<br>Internet Source	<1 %
27	S. Lally, C. Y. Tan, D. Owens, G. H. Tomkin. "Messenger RNA levels of genes involved in dysregulation of postprandial lipoproteins in type 2 diabetes: the role of Niemann–Pick C1-like 1, ATP-binding cassette, transporters G5 and G8, and of microsomal triglyceride transfer protein", Diabetologia, 2006	<1 %

Publication

28	Qinghai Meng, Yu Li, Tingting Ji, Ying Chao et al. "Estrogen prevent atherosclerosis by attenuating endothelial cell pyroptosis via activation of estrogen receptor α-mediated autophagy", Journal of Advanced Research, 2020<br>Publication	<1%

Exclude quotes	On	Exclude matches	Off
Exclude bibliography	On		
