# Supplementary material for: Excessive exogenous cholesterol activating intestinal LXRα-ABCA1/G5/G8 signaling pathway can not reverse atherosclerosis in ApoE−/− mice
Source: Lipids Health Dis. 2023 Apr 15;22:51. doi: 10.1186/s12944-023-01810-6 (PMC10105390; doi:10.1186/s12944-023-01810-6)
Supplement: Supplementary file 5 — Additional file 5. Supply materials-materials and methods. [file 12944_2023_1810_MOESM5_ESM.doc]

**1. Supplymaterials-Materials and methods**

***1.1. Lesion evaluation***

The desired tissue samples were perfused with PBS and fixed in 4% paraformaldehyde for 12 h. Then the specimen was rinsed in running water for 6 h, soaked in double-distilled water for 1 hour, and then placed in 30% sucrose overnight. Taking the mouse aorta as an example, the heart and aorta are embedded in the OCT, placed in a cryostat, and frozen at -25°C for more than 2 h. Cut an 8 µm section from the aortic sinus and place it on a glass slide. The proximal jejunum section was performed as described above, and every five consecutive sections were stained with Oil Red O and HE to evaluate the degree of lipid deposition and lesions. Image Pro Plus software was used to quantify the aorta and proximal jejunum lesion area and lipid deposition area of ApoE-/- mice. Data were expressed as active area ± SEM (n=5).

***1.2. Caco-2 cell culture and model evaluation***

Oil red O staining was performed to assess the formation of lipid accumulation in the cells. In short, the cells were fixed in 4% paraformaldehyde solution for 10 min, and then washed in 60% isopropanol for 15s. Next, under darker conditions, use the freshly prepared Oil Red O working solution to stain at 37°C for 5 min, and then decolorize with 60% isopropanol for 15 seconds. After washing with PBS, the cells were stained with hematoxylin for 5 min and washed with PBS. Observe the stained cells (red) with an optical microscope (Olympus) and acquire an image. And the final concentration of GSK2033 (MedChemExpress, Shanghai, China) for blocking LXRα is 20 nM

***1.3. Methodology for nontargeted lipidomics***

Sample preparation: Pipette 20μL mouse plasma (n=6)or jejunum (n=5)into a centrifuge tube, and add 225μL internal standard (Lyso PE (17:1), SM (17:0), PE (17:0/17:0)) at a concentration of about 5 μg/mL of ice methanol), vortex for 10 s, add 750 μL of MTBE, vortex for 10 s, shake for 10 min at 4°C, add 188 μL of deionized water, vortex for 20 s, and centrifuge at room temperature. Stir at 4°C and 18000 rpm for 2 min, pipette 350 μL of supernatant into a 1.5 mL centrifuge tube, place it in a centrifugal concentrator, and dry for 2 h. Using methanol: toluene, the sample was dissolved in 110 μL of 9:1 solution, vortexed for 15 min, sonicated for 15 min, and then centrifuged at 18000 rpm for 10 min. Put 60 μL of supernatant into an injection bottle and inject it into the machine for analysis. Chromatographic conditions, mass spectrometry conditions, and statistical methods are described in detail in the references. For example, PCA principal components and OPLS-DA partial least squares are obtained through the statistical software (https://www.omicshare.com) and GENE DENOVO Biological Company in Mataboanalysis 3.0 (http://www.metaboanlyst.ca) cloud platform. Discriminant analysis results. Lipid metabolites satisfying P value ≤ 0.05 + fold change ≥ 1.5 or ≤ 0.667 are defined as significantly different lipids.

***1.4. Real-time quantitative polymerase chain reaction (RT-PCR)***

Total RNA from tissues and cells was extracted using Trizol reagent (Thermo Fisher, USA). The NanoDrop ™ One/One C ultra-micro ultraviolet spectrophotometer (Thermo Fisher, USA) was used to detect the purity and concentration of the extracted total RNA. Then, 1 μg of RNA was converted into cDNA by using HiScript II 1st Strand cDNA Synthesis Kit (Vazyme, China), and the cDNA concentration was detected using NanoDrop ™ One. Reactions were performed on the QuantStudio ™ 6 Flex real-time PCR system using the SYBR ™ GREEN fluorescent dye method. The sequences of the RT-PCR primers used are as follows:

NPC1L1, forward 5′-TGAGGGAGGAGTGTGTGCTGTC-3′

reverse 5′-TGGCAGGACTTGAGGAGGTGAG-3′;

ABCG5, forward 5′-CTGAGTCCAGAGGGAGCCAGAG-3′

reverse 5′-CACGGTTGCTGACGCTGTAGG-3′;

ABCG8, forward 5′-CCAACTGCTGCCCAACCTGAC-3′

reverse 5′-GCTCGGCGATTACGTCTTCCAC-3′;

ABCA1, forward 5′- CGTTTCCGGGAAGTGTCCTA-3′

reverse 5′- GCTAGAGATGACAAGGAGGATGGA-3′;

ACC, forward 5′-GCCAGCACACTGAACGATGGAG-3′

reverse 5′-TGGGGTCTACGGCAGCATCAG-3′;

SREBP, forward 5′-CTGGCACCGTTGTCTGGATTGG-3′

reverse 5′-TGGGCTCTGTTCCGTCACCTG-3′;

HMGCR, forward 5′-GCCGTCATTCCAGCCAAGGTG-3′

reverse 5′-TTTGCTGCGTGGGCGTTGTAG-3′;

FAS, forward 5′-AGAAGCTGGCTGGCCTGGTAG-3′

reverse 5′-GCTGCCGCAGTAAGAAGTGGAG-3′;

LXRα, forward 5′-TGAGGGAGGAGTGTGTGCTGTC-3′

reverse 5′-TGGCAGGACTTGAGGAGGTGAG-3′;

LXRβ, forward 5′-TGAGGGAGGAGTGTGTGCTGTC-3′

reverse 5′-TGGCAGGACTTGAGGAGGTGAG-3′;

GAPDH, forward 5′-CGGAGTCAACGGATTTGGTCGTAT-3′

reverse 5′-AGCCTTCTCCATGGTGGTGAAGAC-3′.

The specificity of all PCR products was evaluated by melting curve analysis. Relative gene expression was analyzed using the 2^-ΔΔCt^ method and normalized with GAPDH as an internal control.

***1.5. Western blot analysis***

Jejunum tissue and Caco-2 cells were lysed using RIPA lysate and Phenylmethanesulfonyl fluoride (PMSF; Solarbio Life Sciences, Beijing, China) (100: 1). BCA detection kit (CWBIO, Beijing, China) detects protein concentration. Proteins were then separated on a 10% gel (20μg per lane) using sodium lauryl sulfate-polyacrylamide gel electrophoresis (SDS-PAGE, Solarbio Co., Beijing, China) (100V, 90min). The relevant proteins were then transferred to a 0.45 µm polyvinylidene fluoride membrane (PVDF, Formex, Darmstadt, Germany). Thereafter, the membrane was blocked in Tris-buffered saline solution containing 5% skimmed milk powder and 0.1% Tween-20 (TBS-T) at 20℃ for 2 h, and then blocked with anti-LXRα (Abclonal A3974, Wuhan, China), NPC1L1 (Abclonal A10049, Wuhan, China), ABCG5 (Abclonal A8589, Wuhan, China), ABCG8 (Abclonal A1880, Wuhan, China), ABCA1 (Abclonal A21976, Wuhan, China), and β-actin (Abcam ab8226, Cambridge, UK) was gently shaken at 4℃ overnight. The next day, the membrane was rinsed 3 times with TBS-T (10min each time) and incubated with horseradish peroxidase-conjugated secondary antibody (diluted to 1: 5000, Biosharp, Beijing, China) at room temperature for 2 h. Finally, protein bands were visualized by enhanced chemiluminescence (ECL; Merck Millipore, Darmstadt, Germany) and relative protein levels were quantified using Image Lab software.

***1.6. Immunofluorescence***

Tissue sections or cells were fixed in 4% paraformaldehyde solution for 30 min, TBS-T was rinsed 3 times (10 min each time), the sections were blocked in 5% BSA for 2 h, and then anti-LXRα (Abclonal A3974, Wuhan, China), NPC1L1 (Abclonal A10049, Wuhan, China). Incubate at 20℃ for 2h, rinse with TBS-T 3 times (10min each time), and mix with fluorescent secondary antibody (diluted to 1: 100, CWBIO, Beijing, China) at Incubate for 2 h in 20℃, rinse 3 times with TBS-T (10min each time), incubated with DAPI staining solution for 5 min, and rinse 3 times with TBS-T (10 min each time). Cover film. Five independent experiments were performed.

***1.7. Immunohistochemical***

Jejunum tissue or Caco-2 cells were fixed in 4% paraformaldehyde solution for 30 min, TBS-T was rinsed 3 times (10 min each time), the sections were blocked in 5% BSA for 2 h, and then corresponding antibodies ABCG5 (Abclonal A8589, Wuhan, China), ABCG8 (Abclonal A1880, Wuhan, China) (1: 100 dilution) (diluted to 1: 100) incubated at 20℃ for 2h, rinsed with TBS-T 3 times (10 min each time), and mix with fluorescent secondary antibody (diluted to 1: 100) at Incubate for 2 h in 20℃, rinse 3 times with TBS-T (10 min each time), incubate with DAPI staining solution for 5 min, and rinse 3 times with TBS-T (10 min each time). Mounting with an anti-fluorescence quencher. Five independent experiments were performed.
